# Potato straw incorporation improves soybean agronomic traits and yield components and is associated with shifts in rhizosphere soil properties microbial communities and metabolites

**DOI:** 10.3389/fmicb.2026.1990822

**Published:** 2026-10-14

**Authors:** Anle He, Yulong Wang, Huachun Guo, Jun Li, You Zhang, Danqing Huang, Fang Yang, Rui Zhang

**Affiliations:** College of Agronomy and Biotechnology, Yunnan Agricultural University, Kunming, China

**Keywords:** co-occurrence network, potato straw incorporation, soil metabolome, soil microbial community, soil physicochemical properties, soybean (*Glycine max*)

## Abstract

**Background:**

The soil-improving effects of cereal straw incorporation have been well documented; however, potato straw differs markedly from cereal straw in composition and degradation characteristics, and whether straw return is associated with the soil microbial ecosystem and with gains in yield components through pathways similar to those of cereal straw remains unclear.

**Methods:**

In this study, a field-embedded pot experiment was conducted to characterize the interaction network among soil physicochemical properties, microbial communities, and metabolites under potato straw incorporation, and to elucidate its regulatory effects on soybean growth and yield components.

**Results:**

Compared with no incorporation, straw incorporation significantly increased soil organic matter (by 21.60%), available potassium (35.39%), alkali-hydrolyzable nitrogen (16.94%), and available phosphorus (15.42%), and significantly enhanced the activities of acid phosphatase (28.63%) and polyphenol oxidase (16.04%). Amplicon sequencing identified treatment-associated enrichment of bacterial and fungal taxa previously linked to residue decomposition, nutrient cycling (e.g., *Paenibacillus* and *Nitrosomonadaceae*), or plant-beneficial interactions(e.g., arbuscular mycorrhizal fungi [Glomeromycota] and *Bionectria*). Correlation analysis between the soil metabolome and the microbial community showed that both bacterial and fungal communities exhibited a dichotomous pattern of contrasting metabolite preferences: among bacteria, Proteobacteria-dominated taxa were positively correlated with organic acid metabolites, whereas taxa dominated by Gemmatimonadota, Firmicutes, and Actinobacteria were positively correlated with aromatic amino acids and nitrogen-containing heterocyclic compounds, each group being negatively correlated with the metabolites preferred by the other; among fungi, root-symbiotic taxa were positively correlated with secondary metabolites such as flavonoids, whereas saprotrophic fungi were positively correlated with plant-residue decomposition markers such as 2-furoic acid and geranylgeraniol. Three relatively independent organic nitrogen transformation pathways—arginine metabolism, histidine degradation, and purine nucleoside catabolism–were also detected, and pH, organic matter, urease (S-UE), and soybean isoflavone metabolites (daidzein and genistin) showed strong co-occurrence patterns with other network members.

**Conclusion:**

Soybean plant height, stem diameter, pods per plant, and 100-seed weight significantly increased by 14.93%, 17.77%, 19.10%, and 11.40%, respectively. In summary, potato straw incorporation broadened the soil organic nitrogen substrate spectrum by reshaping the soil physicochemical property–microbe–metabolite interaction network, thereby promoting soybean growth and yield components formation through multi-pathway synergy. These findings provide a theoretical basis for the resource utilization of potato straw.

## 1 Introduction

Yunnan Province is one of the major potato (*Solanum tuberosum* L.) producing regions in China, contributing approximately 10.1% of the national output (Liang et al., 2020). Post-harvest potato straw (above-ground vegetative residues) is generally regarded as agricultural agricultural waste, and its large-scale generation poses considerable challenges for environmental management. Therefore, potato stalk returning could not only enhance the recycling efficiency of natural resources and promote the sustainable development of modern agriculture, but also improve following crop economic efficiency (Nawaz and Kumar, 2023).

As a valuable soil amendment, crop straw incorporation can return a proportion of the nutrients absorbed during crop growth to the soil, thereby improving soil fertility, reducing bulk density (Ibrahim et al., 2020), and facilitating subsequent crop growth (Wang et al., 2021b). Anning et al. (2021) reported that maize straw incorporation significantly improved soil physicochemical properties by increasing soil organic carbon and total nitrogen contents, enhancing soil porosity, and reducing bulk density, which consequently led to higher potato yield and protein accumulation. Long et al. (2023) suggested that the regulation of soil moisture status and the enhancement of nutrient supply capacity through straw incorporation are key driving factors for promoting aboveground biomass accumulation and yield improvement. Furthermore, previous studies have demonstrated that straw incorporation can achieve an overall improvement in soil quality by regulating soil enzyme activities, optimizing soil nutrient balance, and improving soil texture (Lin et al., 2023). For instance, following maize straw incorporation, the microbial biomass carbon content in the rhizosphere soil of subsequent maize and soybean crops increased significantly, accompanied by marked enhancements in the activities of key soil enzymes, including urease, invertase, catalase, and acid phosphatase (Yang et al., 2023b; Cui et al., 2024).

Soil microorganisms serve as pivotal drivers of nutrient cycling and energy transformation within soil ecosystems, and the stability of their community structure and function is essential for maintaining soil ecological quality (Cong et al., 2020). Regarding the response of microbial community structure, the effects of different types of straw incorporation on soil microbial communities vary considerably. Yang et al. (2022) found that wheat straw incorporation exerted a substantially greater regulatory effect on the rhizosphere fungal community of soybean than on the bacterial community, as evidenced by increased relative abundances of cellulolytic fungi (e.g., *Acremonium, Trichoderma*, and *Myrmecridium*), along with elevated proportions of saprotrophic fungi (e.g., *Exophiala*) and nitrogen-cycling bacteria (e.g., *Chryseolinea*), collectively providing a favorable microenvironment for soil nutrient transformation and crop growth. Both Ding and Li (2023) and Wu et al. (2020) confirmed that straw incorporation significantly enhances soil bacterial community diversity, with Proteobacteria, Acidobacteriota, and Chloroflexi identified as the dominant phyla. The optimization of these community structures is of considerable significance for the stability of soil ecological functions. Moreover, straw incorporation can modulate soil metabolite composition by altering microbial activity and root exudate profiles, thereby influencing the material exchange processes between soil and crop systems. Chen et al. (2025) demonstrated that rice straw incorporation significantly affected the composition of both crop root exudates and soil metabolites; notably, with increasing root exudate release, the relative abundances of metabolites such as sorbitol, myristic acid, and pentadecanoic acid were markedly elevated in high-nitrogen soils. Liu et al. (2023b) further clarified that wheat and rice straw incorporation primarily induced alterations in soil metabolic components, including lipids, organic acids, organic nitrogen compounds, and benzenoids, which were highly enriched in lipid metabolism and amino acid metabolism pathways, and were also extensively involved in nucleotide metabolism, xenobiotic metabolism, and secondary metabolism.

In summary, Straw-incorporation research has long focused on gramineous (Poaceae) crops, and whether its conclusions can be extrapolated to solanaceous straw remains unclear. Potato straw differs from gramineous straw in three traits directly relevant to soil processes: it accumulates large amounts of potassium and consequently returns substantially more potassium to the soil through its residues; it has a low C/N ratio and low lignin content, so its incorporation is expected to be associated with nitrogen mineralization rather than immobilization, in contrast to the short-term nitrogen immobilization associated with wheat and rice straw; and it contains steroidal glycoalkaloids with antifungal activity, which may impose selective pressure on the soil fungal community, but their persistence after soil incorporation and their ecological relevance to the rhizosphere remain uncertain. However, whether these compositional differences translate into detectable soil biological responses has not been systematically documented. To fill this gap, in this study we conducted a field-embedded pot experiment and integrated microbial community sequencing with untargeted metabolomics to systematically dissect how potato straw incorporation coordinates shifts in microbial community structure and metabolite profiles to jointly promote soybean growth. Our findings are expected to provide a theoretical basis and practical reference for the efficient resource utilization of potato straw in Yunnan Province and for the subsequent healthy cultivation of soybean.

## 2 Materials and methods

### 2.1 Experimental site description

The experiment was conducted in 2024 at the experimental farm of Yunnan Agricultural University, < city>Kunming < /city>, China (102°42′E, 25°22′N; 1,920 m above sea level). The soil is classified as red soil (Ultisol, USDA Soil Taxonomy). Baseline physicochemical properties of the topsoil (0–20 cm) were as follows: pH 5.34, organic matter 7.93 g kg^−1^, total nitrogen (TN) 0.47 g kg^−1^, total phosphorus (TP) 0.72 g kg^−1^, total potassium (TK) 3.20 g kg^−1^, alkali-hydrolyzable nitrogen (AN) 31.54 mg kg^−1^, available phosphorus (AP) 2.26 mg kg^−1^, and available potassium (AK) 62.42 mg kg^−1^.

### 2.2 Experimental materials

The potato straw used in this experiment was derived from the cultivar ‘Dianshu 1428', and the soybean cultivar employed was ‘Cuilvdou'. The nutrient contents of the potato straw (dry weight basis, water content: 10 %) were as follows: TN 34.5 g kg^−1^, TP 4.3 g kg^−1^, and TK 58.7 g kg^−1^.

### 2.3 Experimental design

A pot experiment was conducted using plastic pots (height: 30 cm; inner diameter: 38 cm). Two treatments were established: soybean cultivation without potato straw incorporation (CK) and soybean cultivation with potato straw incorporation (PSI), each with 30 replicate pots. All pots were buried in the field with their upper rims flush with the ground surface, with a spacing of 1 m between treatments.

Prior to the experiment, freshly harvested potato straw was chopped into approximately 5-cm segments using a straw chopper, thoroughly mixed with the topsoil, and filled into the pots. The straw application rate was based on a field incorporation rate of 45,000 kg ha^−1^, corresponding to approximately 510 g of fresh straw per pot (water content: 80%−90%). Soybean seeds with uniform size and germination rate exceeding 95% were sown at six seeds per pot and thinned to three seedlings per pot at 10 days after emergence. Each pot received 1.5 g of diammonium phosphate (N: 18%; P_2_O_5_: 46%) and 0.75 g of potassium sulfate (K_2_O: 50%) as basal fertilizer, applied by mixing into the topsoil prior to sowing. Throughout the entire growth period, uniform field management practices were implemented, including irrigation, fertilization, and pest and disease control.

### 2.4 Sample collection and yield parameter determination

#### 2.4.1 Measurement of agronomic traits and yield components

At the soybean harvest stage, yield-related parameters including plant height, stem diameter, node number per plant, and 100-seed weight were measured. Plant height was measured from the cotyledon scar to the apical growing point using a straight ruler. Stem diameter was determined at the fifth internode from the base using a vernier caliper. The 100-seed weight was obtained by randomly selecting and weighing 100 seeds on an analytical balance.

#### 2.4.2 Soil sample collection and preservation

Rhizosphere soil samples were collected at the soybean harvest stage. For soil physicochemical property determination, enzyme activity assays, untargeted metabolomics analysis, and microbial community analysis, rhizosphere soil from five pots with uniform plant growth per treatment was pooled to form one composite sample, yielding six biological replicates per treatment. Visible plant debris, animal residues, and gravel were removed from all samples prior to further processing. Soil enzyme activities were measured using fresh soil samples immediately after collection. Each composite sample was subsequently divided into two equal sub-samples: one was stored at −80 °C for microbial community sequencing and metabolomics analyses, and the other was air-dried, ground, and passed through a 0.15-mm mesh sieve for the determination of soil organic matter and other basic physicochemical properties. Rhizosphere soil was sampled only at soybean harvest.

#### 2.4.3 Soil sample analysis

Soil physicochemical properties: alkali-hydrolyzable nitrogen was determined by the alkali diffusion method; available phosphorus by the molybdenum–antimony colorimetric method; available potassium by flame photometry; organic matter by the Walkley–Black dichromate oxidation method; pH by the potentiometric method (water-to-soil ratio of 5:1, v/w); total nitrogen (TN) by the Kjeldahl method; total phosphorus (TP) by NaOH fusion followed by molybdenum–antimony spectrophotometry; and total potassium (TK) by NaOH fusion (Duan et al., 2024). Soil enzyme activities: Urease activity was assayed using the sodium phenol colorimetric method; acid phosphatase activity by the disodium phenyl phosphate colorimetric method; polyphenol oxidase activity by the pyrogallol colorimetric method; and catalase activity by UV spectrophotometry (Duan et al., 2024).

#### 2.4.4 High-throughput sequencing of soil microbial communities

Genomic DNA was extracted from each sample using a standard soil DNA extraction kit, and its quality and integrity were verified by 1% agarose gel electrophoresis and NanoDrop spectrophotometry. For bacterial community analysis, the V3–V4 hypervariable regions of the 16S rRNA gene were amplified using primers 338F (5′-ACTCCTACGGGAGGCAGCAG-3′) and 806R (5′-GGACTACHVGGGTWTCTAAT-3′). For fungal community analysis, the ITS1 region was amplified using primers ITS1F (5′-CTTGGTCATTTAGAGGAAGTAA-3′) and ITS2 (5′-GCTGCGTTCTTCATCGATGC-3′). PCR amplification was performed using TransGen AP221-02 reagents on an Applied Biosystems GeneAmp^®^ 9700 thermal cycler (Thermo Fisher Scientific, Waltham, USA). PCR products were quantified using the QuantiFluor™-ST blue fluorescence quantification system (Promega, Madison, USA), and sequencing libraries were constructed using the TruSeq™ DNA Sample Prep Kit. Paired-end sequencing (2 × 250 bp) was performed on an Illumina MiSeq platform by Majorbio Bio-Pharm Technology Co., Ltd. (Shanghai, China).

Raw sequencing reads were quality-filtered by removing sequences with average quality scores below 20 and reads shorter than 200 bp. Chimeric sequences were identified and removed using UCHIME. The remaining high-quality reads were processed using DADA2 to generate amplicon sequence variants (ASVs). Taxonomic classification of ASVs was performed against the SILVA database (release 138) for bacteria and the UNITE database (version 9.0) for fungi, using a confidence threshold of 70%. Based on ASV representative sequences and abundance profiles, downstream analyses were conducted, including alpha diversity (Sobs index, Chao1 index, Shannon index), taxonomic composition analysis, LEfSe differential species analysis, and Spearman correlation analysis. All bioinformatic analyses were performed on the Majorbio Cloud Platform (www.majorbio.com).

#### 2.4.5 Untargeted metabolomics analysis

Soil metabolomics analysis was conducted by Majorbio Bio-Pharm Technology Co., Ltd. (Shanghai, China). Samples were subjected to cryogenic grinding and centrifugation prior to analysis on an LC-MS platform. The LC-MS analysis was conducted on a Thermo Fisher Scientific UHPLC-Exploris 480 system, comprising an ultra-high-performance liquid chromatograph coupled with a Fourier-transform mass spectrometer.

Chromatographic separation was performed on a Thermo Fisher Scientific UHPLC system coupled to an Orbitrap Exploris 480 mass spectrometer. The analytical column was an ACQUITY UPLC HSS T3 column (100 mm × 2.1 mm, 1.8 μm particle size; Waters, Milford, USA). Mobile phase A consisted of 95% water and 5% acetonitrile (containing 0.1% formic acid, v/v), and mobile phase B consisted of 47.5% acetonitrile, 47.5% isopropanol, and 5% water (containing 0.1% formic acid, v/v). The injection volume was 3 μL and the column temperature was maintained at 40 °C. Mass spectra were acquired in both positive and negative electrospray ionization (ESI) modes over a scan range of m/z 70–1,050, with a resolution of 60,000 FWHM.

Raw data were imported into Progenesis QI (Waters Corporation, Milford, USA) for peak picking, alignment, and normalization. Metabolite identification was achieved by matching precursor masses and MS/MS fragmentation spectra against the Human Metabolome Database (HMDB, http://www.hmdb.ca/), the KEGG Compound Database, and the MassBank database, with a mass error tolerance of ±10 ppm. Multivariate statistical analyses, including partial least squares-discriminant analysis (PLS-DA), were performed to assess overall metabolic differences between treatments. Differentially accumulated metabolites (DAMs) were identified based on variable importance in projection (VIP) > 1, and *p* < 0.05 (Student's *t*-test). KEGG pathway enrichment analysis was performed to identify significantly enriched metabolic pathways (*p* < 0.05).

### 2.5 Definition of differential metabolites and microbial taxa for correlation and co-occurrence network analysis

To identify differential metabolites between treatments, the overall metabolic differences were first visualized using principal component analysis (PCA) and partial least squares discriminant analysis (PLS-DA). Differential metabolites were subsequently screened using orthogonal partial least squares discriminant analysis (OPLS-DA) based on variable importance in projection (VIP) values, in combination with fold change (FC) and *p*-values from univariate analysis (Student's *t*-test). After feature-level quality control and normalization, differential metabolites were defined using OPLS-DA (screening criteria: VIP ≥ 1, and *p* < 0.05). Fold change was used only to determine direction: FC > 1 was defined as upregulated, and FC < 1 as downregulated, without additional fold-change magnitude thresholds. A total of 485 differential metabolites were obtained, comprising 360 upregulated and 125 downregulated metabolites. For ease of presentation, Figure 1B lists the 30 metabolites with the highest VIP values. From the 485 differential metabolites, integrated analysis further retained 25 metabolites that simultaneously had a fragmentation score > 0.65 and were successfully mapped to KEGG pathways. These 25 representative metabolites were selected for subsequent correlation and network analyses.

**Figure 1:**
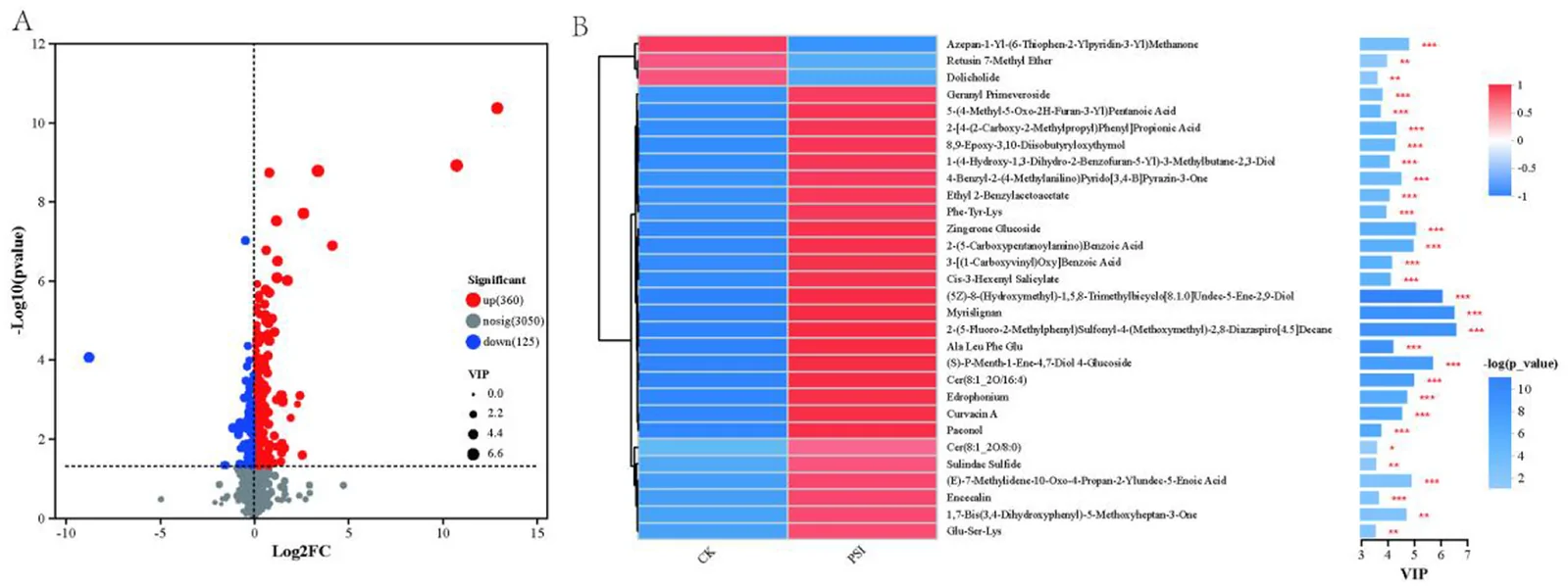
Identification and analysis of differential soil metabolites. **(A)** Volcano plot illustrating the upregulated and downregulated differential metabolites between the PSI and CK treatments. Red and blue dots represent metabolites that simultaneously satisfied VIP ≥ 1, *p* < 0.05, and FC > 1 or FC < 1, respectively; gray dots did not meet all the above criteria. Dot size is proportional to VIP. **(B)** Hierarchical clustering heatmap and variable importance in projection (VIP) scores of the top 30 differential metabolites with the highest VIP values. CK, control (without straw return); PSI, potato straw incorporation.

To identify differentially abundant microbial taxa, linear discriminant analysis effect size (LEfSe) was applied to both bacterial and fungal community data (LDA score > 2.0, *p* < 0.05). The identified differential metabolites and microbial taxa were subsequently subjected to Spearman correlation analysis and co-occurrence network analysis as described in Section 1.6.

### 2.6 Statistical analysis

Data organization and descriptive statistics were performed using Microsoft Excel 2024. Independent-samples *t*-tests and *Z*-score standardization were conducted using SPSS 26.0 (IBM Corp., Armonk, NY, USA). Integrated multi-omics analysis was performed on the Majorbio Cloud Platform (https://cloud.majorbio.com).

#### 2.6.1 Microbial community analysis

Alpha diversity indices (Sobs, Chao1, Shannon) were calculated using Mothur (version 1.30, University of Michigan, Ann Arbor, MI, USA). Principal coordinates analysis (PCoA) based on Bray-Curtis distances was performed using the vegan package in R (version 3.3.1, R Foundation for Statistical Computing, Vienna, Austria), and differences in community composition between groups were assessed by permutational multivariate analysis of variance (PERMANOVA, 999 permutations). Differentially abundant microbial taxa from the phylum to genus level were identified using LEfSe (LDA score > 2.0, *p* < 0.05).

#### 2.6.2 Metabolomics data analysis

OPLS-DA was conducted using the ropls package (version 1.6.2, Bioconductor, Buffalo, NY, USA) in R (version 4.2.0). Venn diagrams were generated using the VennDiagram package in R. Compound classification and metabolic pathway annotation were performed using the KEGG database (accessed August 2023). Pathway enrichment analysis was performed using the scipy.stats module in scipy (Python; Version1.0.0, Python Software Foundation, Wilmington, Delaware, USA). Prior to correlation analysis, microbial genera and metabolites with a mean relative abundance < 0.01% or missing in more than 50% of samples were removed. Spearman correlation heatmaps were constructed using complete-linkage hierarchical clustering based on Euclidean distance, with significance levels annotated (^*^p < 0.05, ^**^p < 0.01, ^***^p < 0.001).

#### 2.6.3 Co-occurrence network analysis

Microbial genera were retained if they were identified as differentially abundant taxa by LEfSe analysis (LDA score > 2.0, FDR-adjusted *p* < 0.05). Metabolites were retained if they passed the quality control and annotation criteria described in Section 2.5. Prior to correlation analysis, microbial genera and metabolites with relative abundance < 0.01% and missing in more than 50% of samples were excluded to reduce noise and spurious correlations. Spearman correlation coefficients were calculated among soil physicochemical properties, enzyme activities, genus-level microbial taxa, and differential metabolites. Edges were retained when |*r*| > 0.95 and *p* < 0.05. Network robustness was assessed by bootstrap resampling (1,000 resamplings of samples) and comparison with randomized null networks (comparing topological metrics such as modularity and average degree). Network topology metrics, including node degree, betweenness centrality, and modularity, were calculated using the igraph package in R. Network visualization was performed using Gephi (version 0.9.2, Gephi Consortium, Paris, France). Mantel tests were performed to assess the correlation between treatment (CK vs. PSI) and the overall rhizosphere metabolite profile, based on Bray-Curtis distance matrices, to strengthen multivariate association inference (Appendix 1).

## 3 Results and analysis

### 3.1 Effects of potato straw incorporation on soybean agronomic traits and yield components

As shown in Table 1, yield-related parameters were compared between the PSI and CK treatments. With the exception of node number, all other parameters exhibited significant differences between the two treatments (*p* < 0.05). Compared with CK, the PSI treatment significantly increased plant height, stem diameter, number of pods per plant, and 100-seed weight by 14.93%, 17.77%, 19.10%, and 11.40%, respectively.

**Table 1:** Effects of potato straw incorporation on soybean agronomic traits and yield components.

Treatment	Plant height (cm)	Stem diameter (mm)	Node number	Number of pods per plant	100-seed weight (g)
CK	40.79 ± 0.51	7.09 ± 0.62	9.33 ± 0.58	22.67 ± 0.58	23.95 ± 0.68
PSI	46.88 ± 1.60^*^	8.35 ± 0.16^*^	10.33 ± 0.58	27.00 ± 2.00^*^	26.68 ± 1.22^*^

CK, control (without straw return); PSI, potato straw incorporation.

Values are presented as the mean ± standard deviation (SD).

An asterisk (^*^) within the same column indicates a significant difference between treatments according to Student's *t*-test (*p* < 0.05).

### 3.2 Changes in soil nutrients and enzyme activities following potato straw incorporation

#### 3.2.1 Changes in soil nutrient contents

As presented in Table 2, potato straw incorporation had significant effects on soil nutrient contents in the soybean cultivation system (*p* < 0.05). Compared with CK, the PSI treatment significantly increased all measured nutrient parameters except TK, with particularly pronounced increases observed in organic matter (21.60%) and available potassium (35.39%). Specifically, the PSI treatment elevated soil pH by 0.48%, and increased TN and TP contents by 7.69 and 8.64%, respectively. Available phosphorus and alkali-hydrolyzable nitrogen contents were also enhanced by 15.42 and 16.94%, respectively.

**Table 2:** Effects of potato straw incorporation on soil physicochemical properties.

Treatment	pH	TN (g/kg)	TP (g/kg)	TK (g/kg)	AN (mg/kg)	AP (mg/kg)	AK (mg/kg)	OM (g/kg)
CK	6.25 ± 0.02	0.52 ± 0.01	0.81 ± 0.02	3.41 ± 0.26	40.68 ± 1.84	2.53 ± 0.11	74.73 ± 0.10	8.98 ± 0.34
PSI	6.28 ± 0.01^*^	0.56 ± 0.01^*^	0.88 ± 0.02^*^	3.77 ± 0.11	47.57 ± 1.96^*^	2.92 ± 0.16^*^	101.18 ± 0.10^*^	10.92 ± 0.40^*^

CK, control (without straw return); PSI, potato straw incorporation; TN, total nitrogen; TP, total phosphorus; TK, total potassium; AN, alkali-hydrolyzable nitrogen; AP, available phosphorus; AK, available potassium; OM, organic matter.

Values are presented as the mean ± standard deviation (SD).

An asterisk (^*^) within the same column indicates a significant difference between treatments according to Student's *t*-test (*p* < 0.05).

#### 3.2.2 Changes in soil enzyme activities

As illustrated in Table 3, soil enzyme activities were altered following potato straw incorporation. Compared with CK, the activities of acid phosphatase and polyphenol oxidase in the PSI treatment were significantly higher (*p* < 0.05), with increases of 28.63 and 16.04%, respectively. Urease and catalase activities also increased under the PSI treatment (by 8.33 and 5.66 percentage points); however, the differences were not statistically significant.

**Table 3:** Effects of potato straw incorporation on soil enzymatic activities.

Treatment	S-UE (U/g)	S-ACP (U/g)	S-CAT (U/g)	S-PPO (U/g)
CK	0.12 ± 0.00	7,812.60 ± 143.96	6.01 ± 0.20	3.99 ± 0.24
PSI	0.13 ± 0.01	10,049.08 ± 81.39^*^	6.35 ± 0.12	4.63 ± 0.18^*^

CK, control (without straw return); PSI, potato straw incorporation; S-UE, soil urease; S-PPO, soil polyphenol oxidase; S-ACP, soil acid phosphatase; S-CAT, soil catalase.

Values are presented as the mean ± standard deviation (SD).

An asterisk (^*^) within the same column indicates a significant difference between treatments according to Student's t-test (*p* < 0.05).

### 3.3 Changes in soil microbial community structure following potato straw incorporation

#### 3.3.1 Changes in soil microbial alpha diversity

As shown in Table 4, the Good's coverage values for both bacterial and fungal communities exceeded 99% across all soil samples following potato straw incorporation, indicating that the sequencing depth was sufficient to capture the true composition of the microbial communities.

**Table 4:** Differences in α-diversity of soil bacteria and fungi.

Treatment		Sobs	Chao	Shannon	Simpson	Coverage
Bacteria	CK	1,257.000 ± 53.113	1,263.656 ± 54.692	5.887 ± 0.295	0.006 ± 0.002	0.999 ± 0.000
	PSI	1,414.667 ± 74.056^*^	1,418.152 ± 72.974^*^	6.246 ± 0.138	0.005 ± 0.001	0.999 ± 0.000
Fungi	CK	321.000 ± 67.550	326.761 ± 65.558	3.433 ± 0.630	0.098 ± 0.069	0.999 ± 0.000
	PSI	383.000 ± 43.920	386.212 ± 43.775	3.393 ± 0.377	0.113 ± 0.036	0.999 ± 0.000

CK, control (without straw return); PSI, potato straw incorporation.

Values are presented as the mean ± standard deviation (SD).

An asterisk (^*^) within the same column indicates a significant difference between treatments according to Student's t-test (*p* < 0.05).

For the bacterial community, compared with the no-straw incorporation (CK) treatment, the Sobs and Chao indices were significantly higher in the straw incorporation (PSI) treatment (*P* < 0.05), indicating that straw incorporation significantly increased soil bacterial species richness. The Shannon index was slightly higher, and the Simpson index was slightly lower in the PSI treatment, but neither difference reached statistical significance, suggesting that the evenness and dominance concentration of the bacterial community did not fundamentally change. For the fungal community, compared with CK, the Sobs, Chao, Shannon, and Simpson indices all showed minor numerical differences under the PSI treatment, but none of these differences were statistically significant (*P* > 0.05). This indicates that, under the conditions of this experiment, potato straw incorporation had no significant effect on the alpha diversity of the soil fungal community.

#### 3.3.2 Changes in β-diversity and species composition of soil microbial communities

As shown in Figure 2A, the PSI and CK treatments harbored 2,846 and 2,666 bacterial ASVs, respectively. Shared bacterial ASVs accounted for 19.85% of the total, while treatment-specific ASVs constituted 42.03 and 38.12% for PSI and CK, respectively. Similarly, as shown in Figure 2B, the PSI and CK treatments contained 797 and 645 fungal ASVs, respectively, with shared fungal ASVs representing 19.97% of the total, and treatment-specific ASVs accounting for 46.34 and 33.69% for PSI and CK, respectively. PCoA analysis based on Bray-Curtis distance matrix revealed that the CK and PSI treatments showed a certain separation trend in both bacterial (Figure 2C) and fungal (Figure 2D) communities. PERMANOVA test indicated that the overall community structure differences between the two groups were not statistically significant for both bacteria (*R*^2^ = 0.397, *P* = 0.1) and fungi (*R*^2^ = 0.581, *P* = 0.1). These results collectively indicate that potato straw incorporation promotes an increase in soil microbial species richness and compositional diversity.

**Figure 2:**
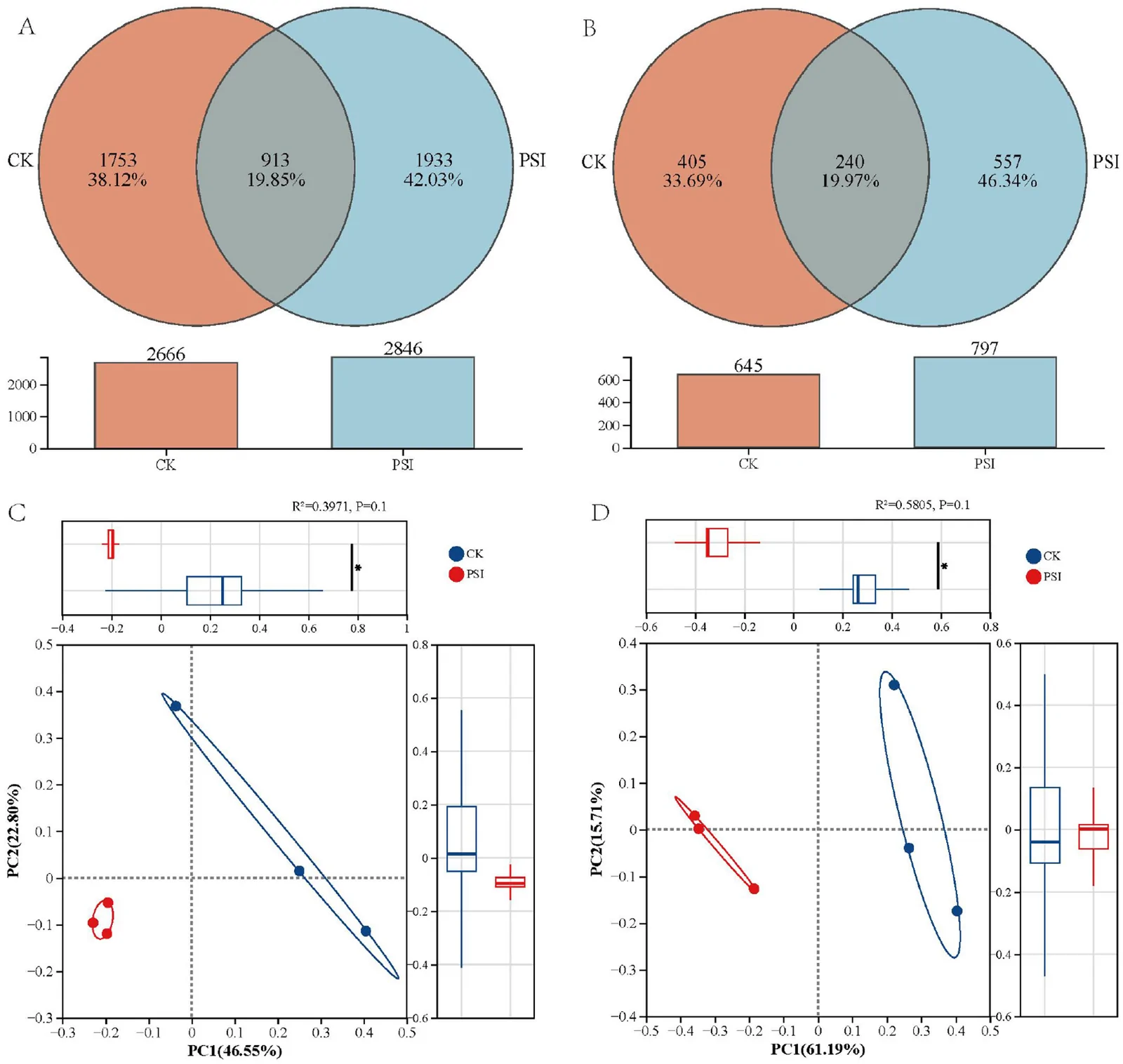
Soil microbial community composition and structure. **(A)** Venn diagram of the bacterial community at the ASV level. **(B)** Venn diagram of the fungal community at the ASV level. **(C)** Principal coordinates analysis (PCoA) of the bacterial community based on Bray-Curtis distance matrix. **(D)** PCoA of the fungal community based on Bray-Curtis distance matrix. CK, control (without straw return); PSI, potato straw incorporation.

#### 3.3.3 Differences in bacterial and fungal community composition at the phylum level

The community bar charts illustrate the phylum-level composition and relative abundances of bacterial (Figure 3A) and fungal (Figure 3B) communities in the soil following potato straw incorporation (Figure 3). For bacterial phyla, compared with CK, the PSI treatment significantly increased the relative abundances of Acidobacteriota (by 3.74 percentage points), Gemmatimonadota (by 1.99 percentage points), Verrucomicrobiota (by 1.37 percentage points), and Firmicutes (by 2.04 percentage points), while significantly decreasing those of Bacteroidota (by 4.78 percentage points) and Cyanobacteria (by 5.65 percentage points). For fungal phyla, the relative abundance of Chytridiomycota significantly increased from 0.95 to 2.84%, and Glomeromycota from 2.74 to 5.12% following straw incorporation, whereas Basidiomycota significantly decreased from 8.47 to 5.07%.

**Figure 3:**
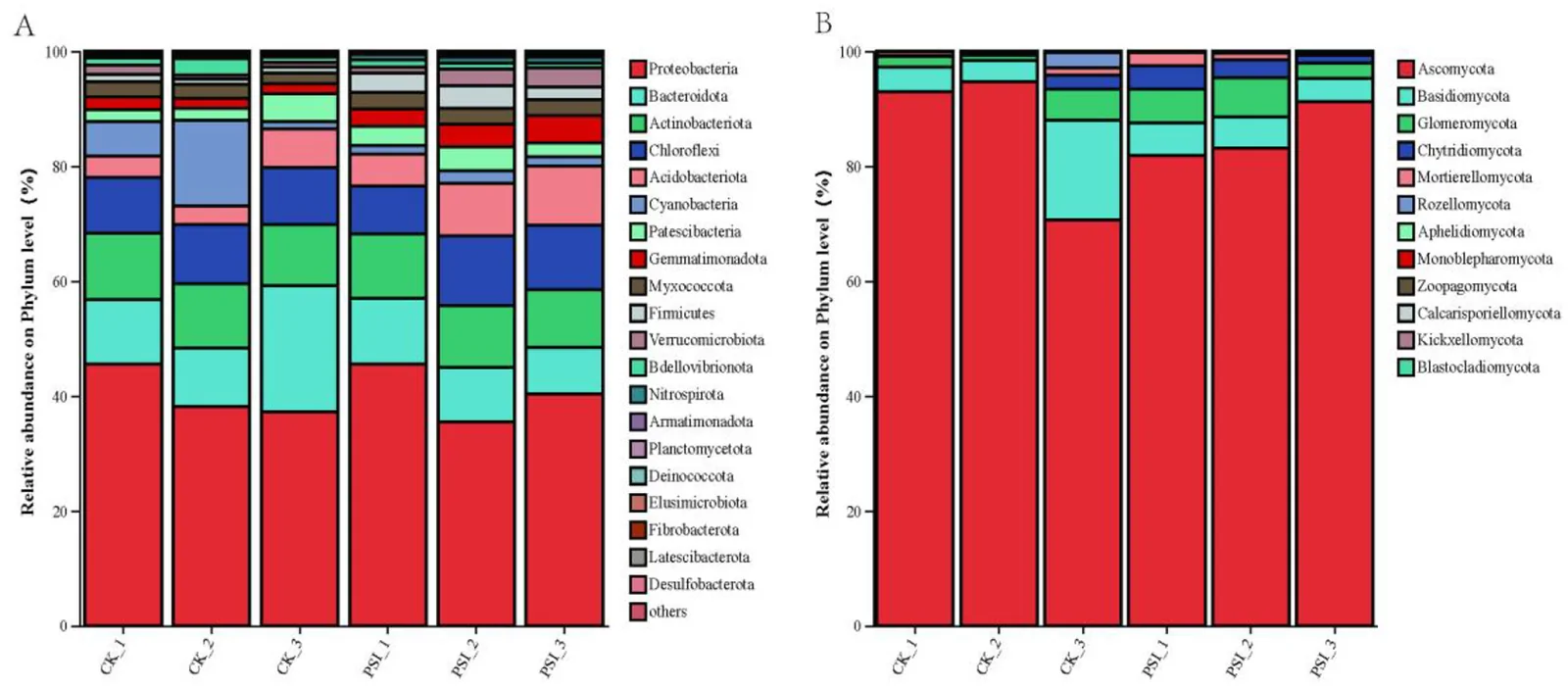
Taxonomic compositions of soil microbial communities at the phylum level. **(A)** Relative abundances of dominant bacterial phyla. **(B)** Relative abundances of dominant fungal phyla. CK, control (without straw return); PSI, potato straw incorporation.

#### 3.3.4 Identification of differential microbial biomarkers via LEfSe analysis

As shown in Figure 4, LEfSe analysis was applied to high-throughput sequencing data, and the results are displayed as a cladogram in a circular layout to illustrate the phylogenetic relationships and abundance variations among different microbial taxa. In the bacterial community (Figure 4A), straw incorporation significantly enriched taxa putatively associated with straw degradation, spanning from the phylum Firmicutes through the class Bacilli to the genus *Paenibacillus*. Additionally, taxa involved in carbon, nitrogen, and phosphorus cycling were significantly enriched, including lineages from Gemmatimonadota to *Gemmatimonas*, as well as Nitrosomonadaceae, Anaeromyxobacteraceae, and *Anaeromyxobacter*. LEfSe identified taxa that differed in relative abundance between treatments. Several PSI-enriched taxa, including *Paenibacillus* and members of Nitrosomonadaceae, have been associated in previous studies with residue transformation or nutrient cycling. These functions were not measured directly in the present experiment. In the CK treatment, taxa primarily associated with recalcitrant organic matter decomposition and carbon stress response were predominantly enriched. These included *Massilia, Aquabacterium*, Xanthomonadaceae, and the *Allorhizobium–Neorhizobium–Pararhizobium–Rhizobium* complex within Proteobacteria. Meanwhile, Cyanobacteriales and Phormidiaceae within Cyanobacteria, as well as Arcticibacter, Parasegetibacter, and Lacibacter within Bacteroidota, were also significantly enriched in the CK treatment.

**Figure 4:**
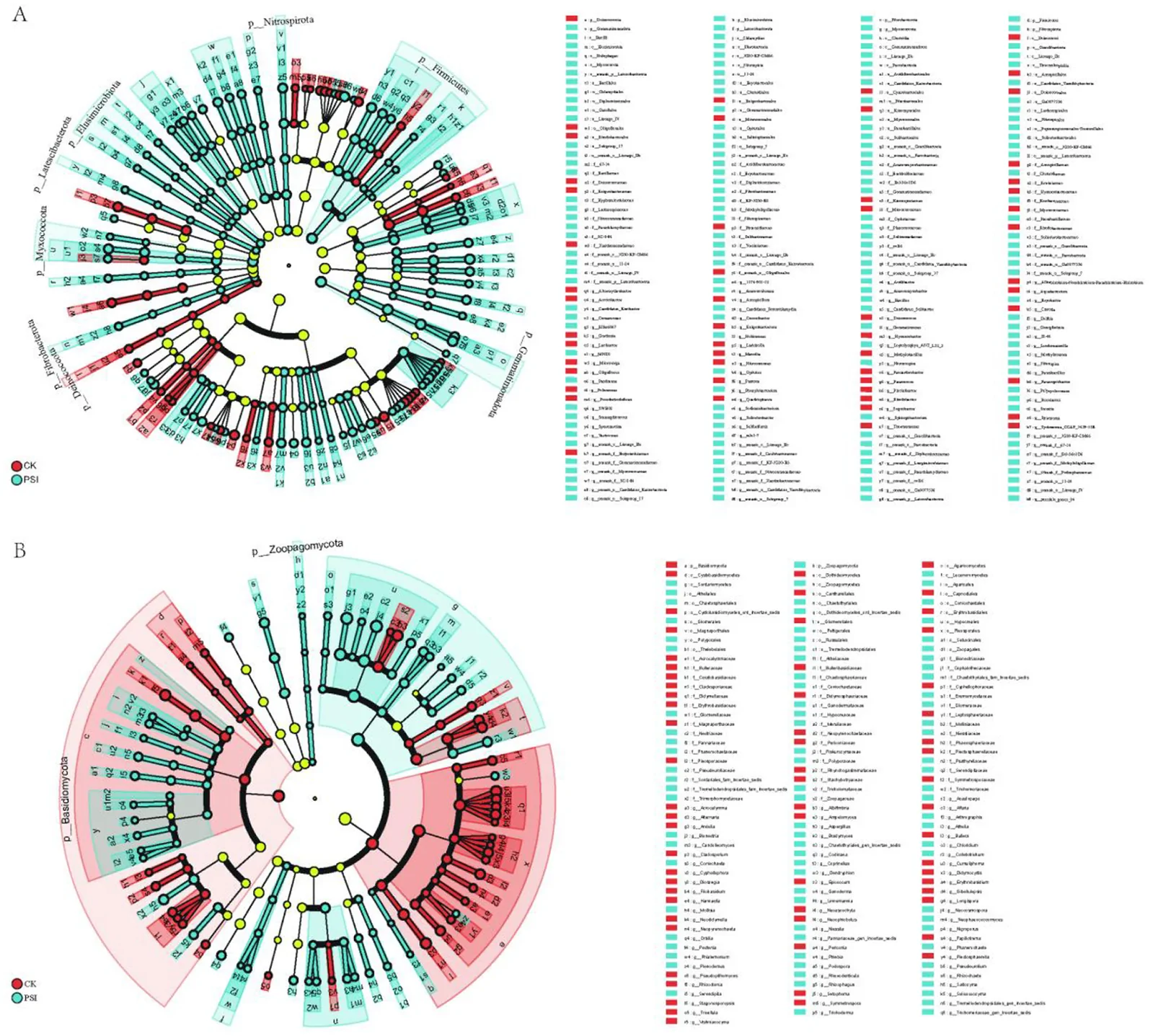
Cladogram indicating the phylogenetic distribution and abundance variations of soil microbial taxa identified by linear discriminant analysis effect size (LEfSe). **(A)** Bacterial community; **(B)** Fungal community. Nodes with different colors represent microbial taxa that are significantly enriched in the corresponding groups and contribute significantly to the observed differences between treatments. Pale yellow nodes indicate microbial taxa that exhibit no significant differences between groups or have negligible impact on group differentiation. CK, control (without straw return); PSI, potato straw incorporation.

In the fungal community (Figure 4B), potato straw incorporation led to the significant enrichment of taxa dominated by arbuscular mycorrhizal fungi (AMF) and Sordariomycetes with biocontrol potential. Specifically, these included Glomerales, Glomeraceae, and Rhizophagus among the AMF, as well as Sordariomycetes, Hypocreales, Bionectriaceae, Bionectria, and Nectriaceae within Ascomycota. In contrast, the CK treatment was predominantly characterized by the enrichment of saprotrophic and potentially pathogenic fungal taxa. These included saprotrophic taxa such as Didymellaceae, Pleosporales, Dothideomycetes, Cladosporium, Cladosporiaceae, and Capnodiales, as well as potentially pathogenic taxa such as Cumuliphoma, Rhizoctonia, Ceratobasidiaceae, Cantharellales, Stachybotryaceae, and Glomerellales.

### 3.4 Changes in soil differential metabolites following potato straw incorporation

#### 3.4.1 Differences in soil metabolite composition

The PLS-DA scores plot (Figure 5A) revealed clear separation between the potato straw incorporation treatment (PSI) and the non-incorporation treatment (CK) in terms of overall soil metabolite composition, with samples from each treatment clustering distinctly on opposite sides of the coordinate axes. This separation indicates that potato straw incorporation substantially altered the overall metabolic profile of rhizosphere soil. Component 1 and Component 2 explained 23.10 and 23.40% of the total variance, respectively.

**Figure 5:**
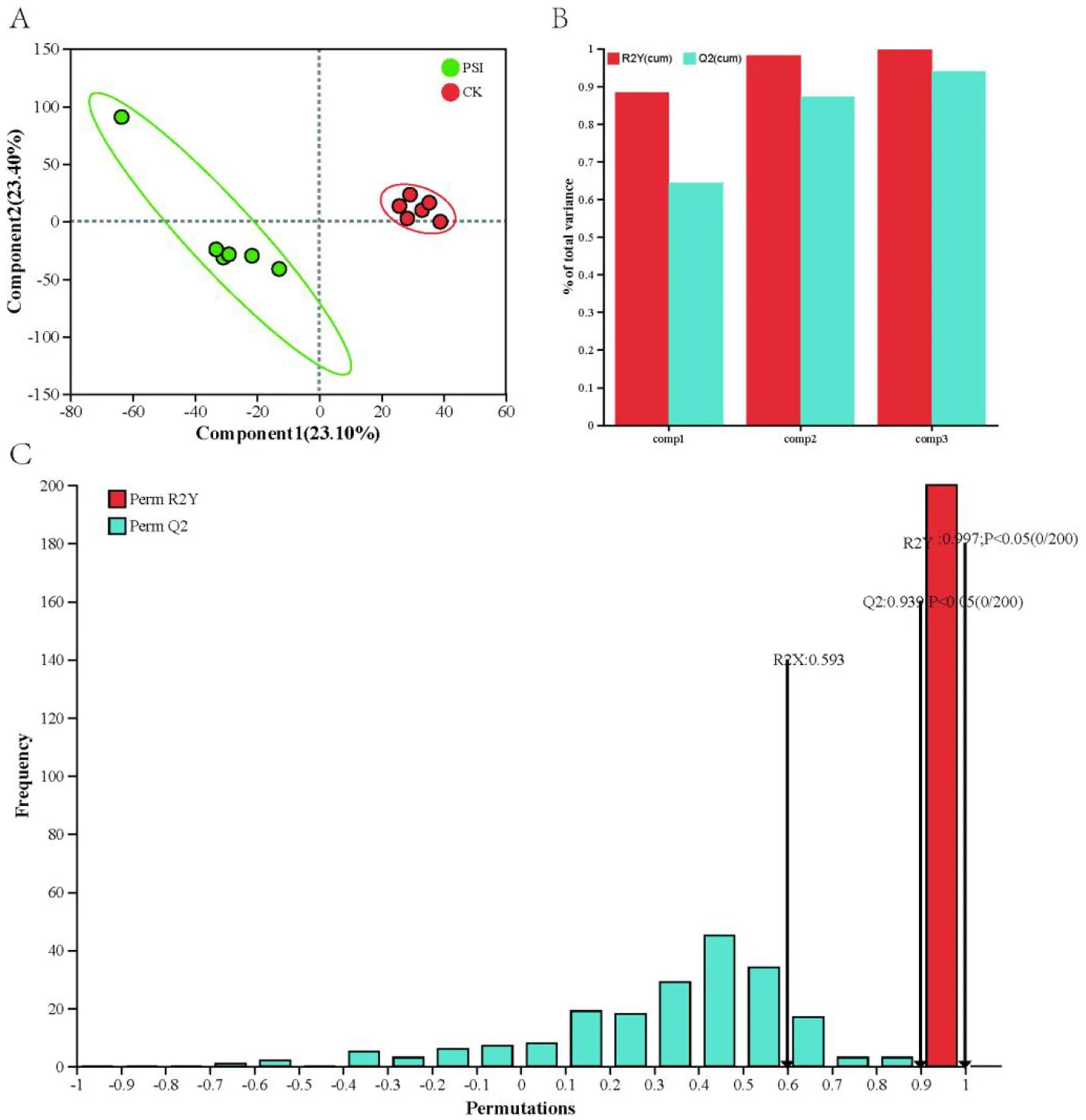
PLS-DA analysis of differential soil metabolites. **(A)** PLS-DA scores plot; **(B)** Model overview showing cumulative *R*^2^*Y* and *Q*^2^ for each component; **(C)** Permutation test results (200 permutations), with annotated values of *R*^2^*Y* = 0.997, *Q*^2^ = 0.939, *R*^2^*X* = 0.593, and *P* < 0.05. PSI: potato straw incorporation treatment; CK: non-incorporation control treatment.

The model overview (Figure 5B) demonstrated that both *R*^2^*Y* (cum) and *Q*^2^ (cum) increased progressively with the number of components (comp 1 to comp 3), reaching approximately 0.997 and 0.939 at the third component, respectively, indicating strong model fit and predictive capability. The validity of the PLS-DA model was further confirmed by permutation testing (200 permutations). The *R*^2^*Y* and *Q*^2^ values of the actual model were 0.997 (*P* < 0.05; 0 out of 200 permutations exceeded the true model value) and 0.939 (*P* < 0.05; 0 out of 200 permutations exceeded the true model value), respectively, with *R*^2^*X* = 0.593. The permuted *Q*^2^ values were broadly distributed well below the true *Q*^2^ value, with no permuted value exceeding it, confirming the absence of overfitting and demonstrating that the observed metabolic differences between treatments reflect genuine biological variation rather than statistical artifacts.

#### 3.4.2 Identification and analysis of soil differential metabolites

Differential metabolites between the two treatments were screened using PCA and OPLS-DA, and the results were visualized as a volcano plot. As shown in Figure 1A, the PSI treatment resulted in the upregulation of 360 metabolites, including lignans (e.g., myrislignan), phenolic glycosides (e.g., zingerone glucoside), and flavonoids (e.g., retusin 7-methyl ether), while 125 metabolites, primarily organoheterocyclic compounds, were downregulated. Based on the VIP values derived from the OPLS-DA model, hierarchical clustering analysis of the top 30 ranked metabolites revealed that 27 differential metabolites were significantly upregulated and three were significantly downregulated in the PSI treatment compared with CK.

#### 3.4.3 Classification of soil differential metabolites and functional pathway enrichment

To characterize the classification of rhizosphere metabolites following straw incorporation, differential metabolites were annotated using the KEGG and HMDB databases. Based on the KEGG database, the annotated metabolites were classified into eight major categories: alkaloids, phenylpropanoids, polyketides, shikimate/acetate-malonate pathway-derived compounds, terpenoids, amino acid-related compounds, fatty acid-related compounds, and flavonoids (Figure 6A). At the secondary classification level, the top three subcategories by metabolite count were diterpenoids (C20), isoflavonoids, and flavonoids, comprising 8, 6, and 6 metabolites, respectively. Annotation against the HMDB database revealed 13 major categories encompassing 310 metabolites (Figure 6B). Among these, lipids and lipid-like molecules, organic acids and derivatives, and organoheterocyclic compounds were the three most abundant categories, accounting for 30.65%, 17.10%, and 14.19% of the total annotated metabolites, respectively.

**Figure 6:**
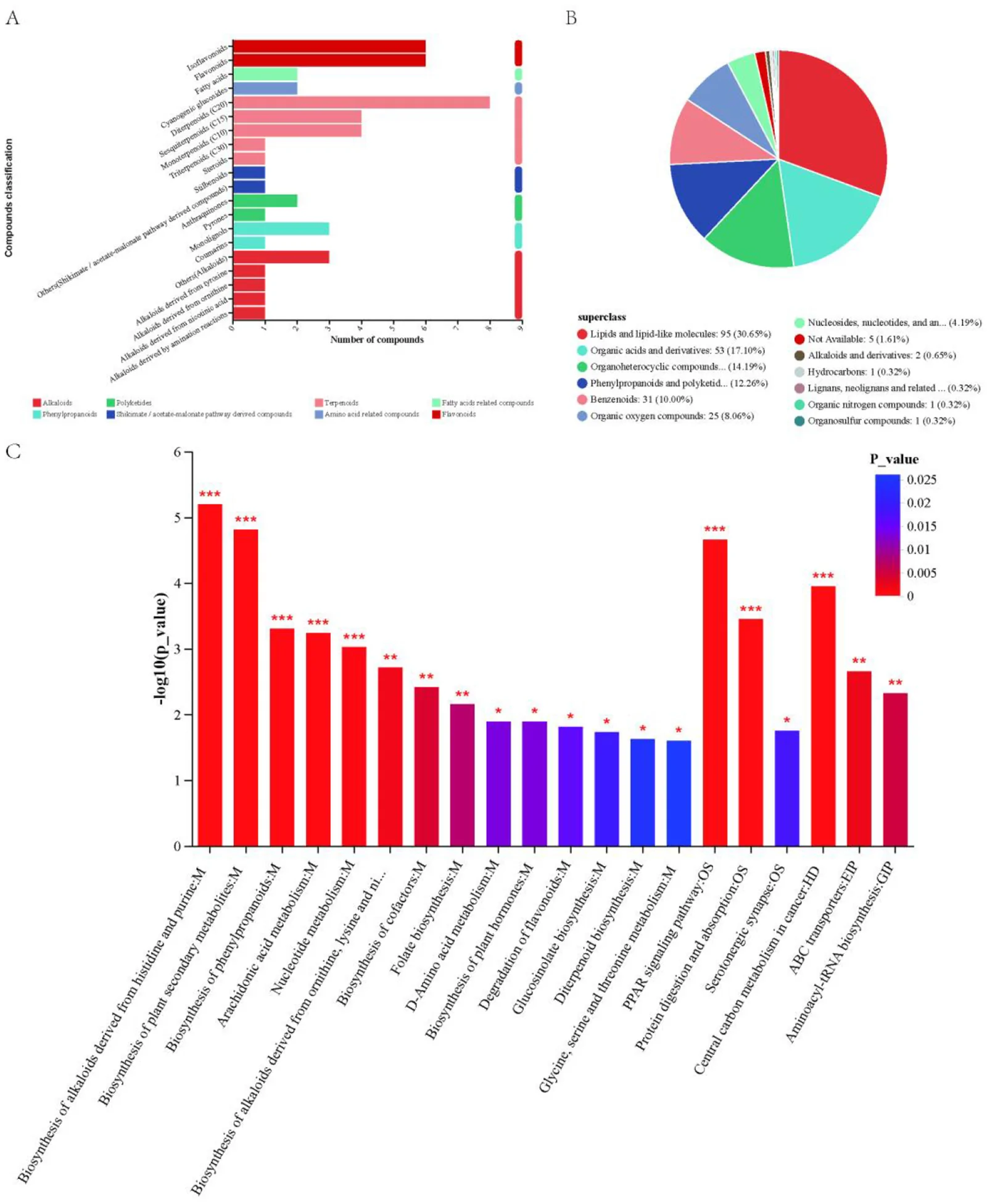
Taxonomic classification and functional pathway enrichment analysis of differential soil metabolites. **(A)** KEGG classification. **(B)** HMDB classification. **(C)** Functional pathway enrichment analysis. CK, control (without straw return); PSI, potato straw incorporation.

As illustrated in Figure 6C, KEGG pathway enrichment analysis was performed on the annotated differential metabolites using a hypergeometric distribution test to identify significantly enriched metabolic pathways. The results revealed that several pathways were significantly enriched, including biosynthesis of alkaloids derived from histidine and purine, biosynthesis of plant secondary metabolites, arachidonic acid metabolism, and biosynthesis of phenylpropanoids.

### 3.5 Integrated analysis of soil physicochemical properties, microbial communities, and metabolites following potato straw incorporation

#### 3.5.1 Correlation analysis between soil metabolites and microbial communities

Spearman correlation analysis was conducted between the top 20 ranked differential metabolites and microbial taxa. The resulting correlation heatmaps exhibited distinct modular clustering patterns (Figure 7).

**Figure 7:**
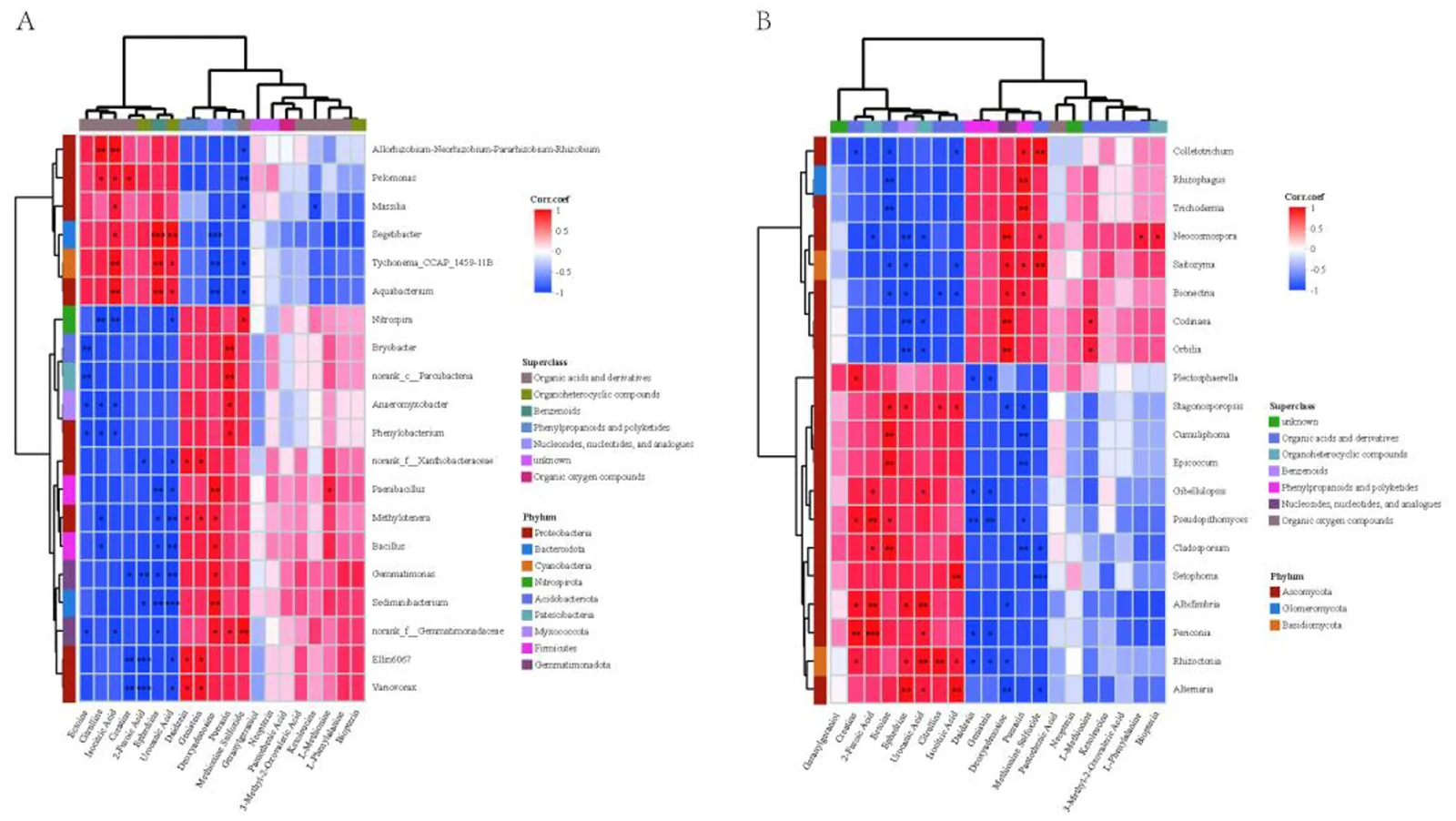
Spearman's rank correlation analysis between the top 20 differential metabolites and dominant microbial community. **(A)** Correlation heatmap between bacterial genera and differential metabolites. **(B)** Correlation heatmap between fungal genera and differential metabolites. The color scale represents the Spearman correlation coefficient (rho), ranging from blue (−1) to red (+1). The top annotation color bar indicates metabolite superclasses, and the left annotation color bar indicates the microbial phylum. Asterisks denote statistical significance: *p < 0.05, **p < 0.01, and ***p < 0.001.

Bacterial community–metabolite correlations: Hierarchical clustering of bacterial genera (row clustering) revealed two major clusters: Cluster 1, dominated by Proteobacteria-affiliated genera, and Cluster 2, primarily composed of genera affiliated with Gemmatimonadota, Firmicutes, and Actinobacteriota. Column clustering of metabolites also identified two categories: Category 1, mainly comprising organic oxygen-containing compounds and organic acid derivatives, and Category 2, predominantly consisting of aromatic amino acids and nitrogen-containing heterocyclic compounds.

Cluster 1 exhibited predominantly significant positive correlations with Category 1 metabolites (*p* < 0.05). Notably, *Segetibacter* showed the strongest correlation with ephedrine (*p* < 0.001), and the *Allorhizobium–Neorhizobium–Pararhizobium–Rhizobium* complex and *Pelomonas* were significantly correlated with citrulline and isocitric acid. Cluster 2 showed significant positive correlations with Category 2 metabolites (*p* < 0.05). Specifically, *Bryobacter* and norank_c__Parcubacteria were significantly correlated with puerarin; *Paenibacillus* and *Sediminibacterium* with deoxyadenosine; and norank_f__Gemmatimonadaceae with methionine sulfoxide (*p* < 0.01 for all). Furthermore, the two bacterial clusters displayed contrasting correlation patterns with the metabolite categories: Cluster 1 was significantly negatively correlated with Category 2 metabolites, while Cluster 2 showed significant negative correlations with Category 1 metabolites. For example, the *Allorhizobium–Neorhizobium–Pararhizobium–Rhizobium* complex, *Pelomonas*, and *Massilia* were significantly negatively correlated with methionine sulfoxide (*p* < 0.05), while *Segetibacter* exhibited a highly significant negative correlation with deoxyadenosine (*p* < 0.001).

Fungal community–metabolite correlations: similarly, hierarchical clustering of fungal genera and metabolites identified two core groups along both the row and column axes. Row clustering divided fungal genera into two major branches: Branch 1, represented by Ascomycota genera such as *Colletotrichum, Trichoderma*, and *Neocosmospora*, with a minor proportion of Glomeromycota taxa; and Branch 2, centered on Ascomycota genera including *Plectosphaerella, Epicoccum*, and *Alternaria*, along with several Basidiomycota taxa. Column clustering classified metabolites into two categories: Category I, mainly consisting of terpenoids (e.g., geranylgeraniol) and organic acid derivatives (e.g., 2-furoic acid); and Category II, predominantly comprising aromatic amino acids (e.g., L-phenylalanine) and flavonoids (e.g., daidzein).

In Branch 1, *Colletotrichum, Rhizophagus, Trichoderma, Neocosmospora, Codinaea*, and *Orbilia* showed significant positive correlations with Category II metabolites, including deoxyadenosine, puerarin, and methionine sulfoxide (*p* < 0.01). Additionally, *Codinaea* and *Orbilia* were significantly positively correlated with L-methionine (*p* < 0.05). Branch 2 exhibited significant positive correlations with Category I metabolites (*p* < 0.05). Specifically, *Cladosporium* was significantly positively correlated with 2-furoic acid and ectoine (*p* < 0.05); *Epicoccum* was positively correlated with geranylgeraniol and creatine, with a highly significant correlation with ectoine (*p* < 0.01); *Alternaria* was significantly correlated with ephedrine, urocanic acid, and isocitric acid (*p* < 0.05); and *Pseudopithomyces* showed significant positive correlations with creatine, 2-furoic acid, and ectoine (*p* < 0.05). Moreover, the two fungal branches exhibited reciprocal negative correlation patterns with the metabolite categories (*p* < 0.05). Branch 1 showed significant negative correlations with Category I metabolites; for instance, *Trichoderma* was negatively correlated with ectoine (*p* < 0.01), and *Neocosmospora* with 2-furoic acid, ephedrine, and urocanic acid (*p* < 0.05), while *Colletotrichum* and *Rhizophagus* were negatively correlated with ectoine (*p* < 0.05). Conversely, Branch 2 displayed significant negative correlations with Category II metabolites; for example, *Epicoccum, Cumuliphoma*, and *Cladosporium* were significantly negatively correlated with puerarin (*p* < 0.01).

#### 3.5.2 Co-occurrence network analysis of soil physicochemical properties, microbial communities, and metabolites

As shown in Figure 8, the co-occurrence network exhibited a complex interaction structure characterized by multiple modules and high connectivity. In the bacterial co-occurrence network, pH was identified as a highly connected node, densely connected to organic matter (OM), *Sediminibacterium*, and the metabolites xanthosine and urocanic acid through both positive and negative edges. Specifically, pH was significantly positively correlated with xanthosine and *Sediminibacterium*, while significantly negatively correlated with urocanic acid. Total nitrogen (TN), alkali-hydrolyzable nitrogen (AN), and available phosphorus (AP) exhibited high degree centrality and were strongly correlated with citrulline, Pseudorhodobacter, and *Nitrospira. Nitrospira* was positively correlated with AN and total potassium (TK) and negatively correlated with L-histidine. *Pseudorhodobacter* was positively correlated with citrulline and negatively correlated with catalase activity (S-CAT), AP, and TN. Urease activity (S-UE) and polyphenol oxidase activity (S-PPO) also exhibited high degree centrality in the network. S-UE formed a positively correlated cluster with the flavonoid metabolites daidzein and genistein, as well as norank_f__Diplorickettsiaceae. S-PPO was negatively correlated with *Hymenobacter*. TK and available potassium (AK) were associated with L-histidine, daidzein, genistein, and several bacterial taxa including *Nitrospira*, norank_f__Pedosphaeraceae, and norank_f__Diplorickettsiaceae. *Pelomonas* was positively correlated with total phosphorus (TP). Ephedrine showed a positive correlation with Segetibacter and a negative correlation with norank_o__Subgroup_17.

**Figure 8:**
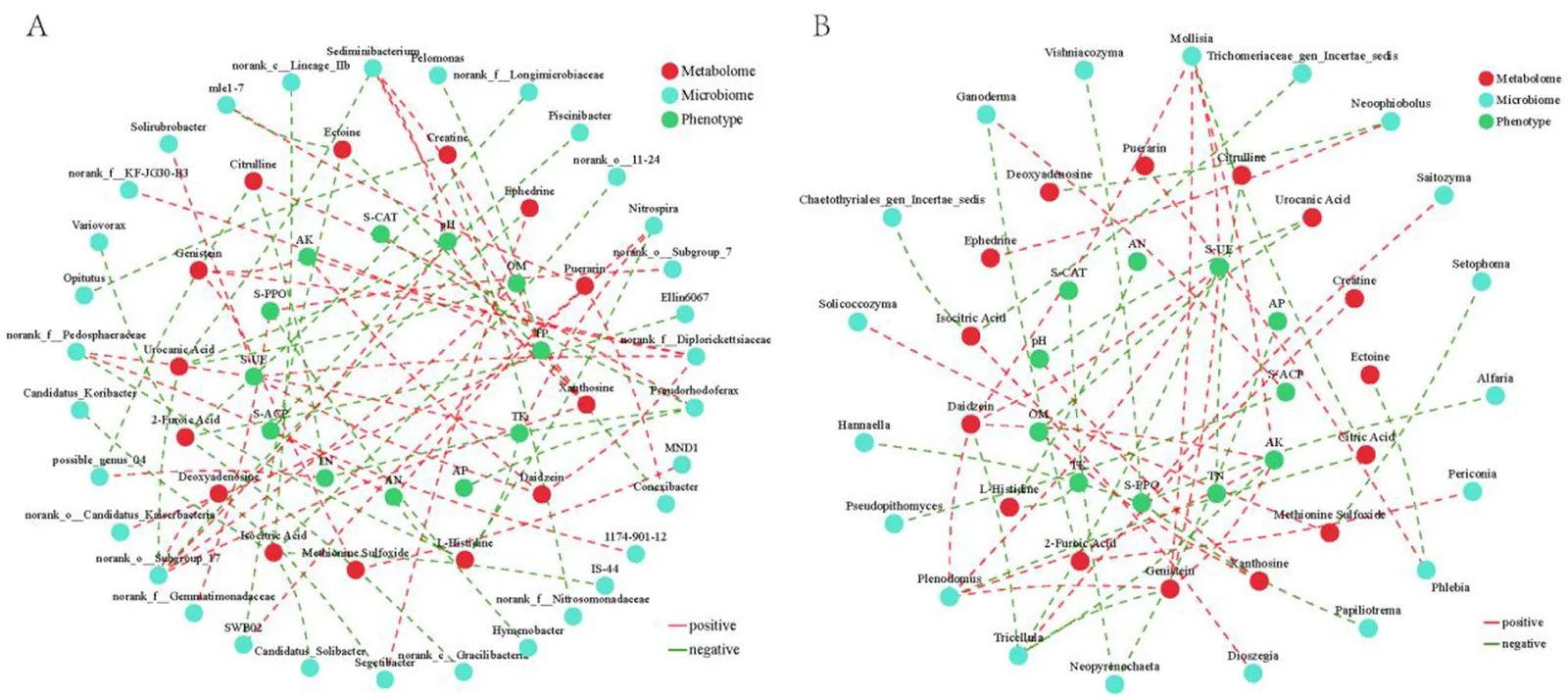
Exploratory co-occurrence networks among soil properties, enzyme activities, microbial genera, and selected metabolites. **(A)** Co-occurrence network of the bacterial community. **(B)** Co-occurrence network of the fungal community. Node size is proportional to the degree of connectivity. Different node colors represent distinct categories: red, blue, and green nodes represent metabolites, microbial genera, and soil properties or enzyme activities, respectively. Solid red lines and dashed green lines indicate positive and negative Spearman correlations, respectively, and edge width is proportional to |rho|. TN, total nitrogen; TP, total phosphorus; TK, total potassium; AN, alkali-hydrolyzable nitrogen; AP, available phosphorus; AK, available potassium; OM, organic matter; S-UE, soil urease; S-PPO, soil polyphenol oxidase; S-ACP, soil acid phosphatase; S-CAT, soil catalase.

In the fungal-metabolite co-occurrence network, S-UE and AK exhibited high degree centrality among the soil variables, whereas daidzein and genistein exhibited high degree centrality among the metabolite nodes. These central nodes were densely connected to fungal genera including *Plenodomus, Mollisia*, and *Tricellula* through both positive and negative edges. S-UE and AK were significantly positively correlated with daidzein, genistein, and *Plenodomus*, while *Mollisia* was negatively correlated with citric acid. pH and OM constituted another core phenotypic module, closely linked to xanthosine and urocanic acid. pH was positively correlated with both OM and xanthosine, while negatively correlated with urocanic acid. *Periconia* was positively correlated with 2-furoic acid. S-PPO, as a core phenotypic node, was positively correlated with the yeast genus *Saitozyma*, while exhibiting negative correlations with *Papiliotrema, Alfaria, Hannaella*, and *Vishniacozyma*. Catalase activity (S-CAT) was negatively correlated with *Neopyrenochaeta*. Acid phosphatase activity (S-ACP) was positively correlated with *Ganoderma* and negatively correlated with Pseudopithomyces.

## 4 Discussion

### 4.1 Effects of potato straw incorporation on rhizosphere nutrient content and enzyme activities

Returning straw to the field not only optimizes soil structure and enhances water retention capacity, but also significantly improves nutrient use efficiency (Ding et al., 2025). The abundant carbon, nitrogen, phosphorus, and potassium elements it contains are gradually released as soluble nutrients through microbial decomposition following incorporation, directly replenishing the soil nutrient pool (Li et al., 2024). In the present study, the most pronounced increases were observed in soil organic matter (21.60%) and available potassium (35.39%) following potato straw incorporation. This may be attributed to the significant accumulation of humus and related substances produced during straw decomposition, which substantially elevated soil organic matter content. As a “reservoir” of soil nutrients, organic matter is closely associated with enhanced nutrient availability through adsorption and complexation, and may contribute to the activation and release of potassium.(Xiong et al., 2025). Furthermore, a slight increase in soil pH (0.48%) was observed, which is likely associated with alkaline substances generated during straw decomposition, facilitating nutrient transformation and uptake by soybean, and ultimately contributing to yield improvement (Zhang et al., 2024).

Soil enzymes, functioning as “catalysts” of soil ecological processes, directly reflect the efficiency of soil nutrient transformation and the intensity of microbial metabolic activity (Li W. et al., 2023). In the present study, straw incorporation significantly increased the activities of acid phosphatase (by 28.63%) and polyphenol oxidase (by 16.04%). This may be attributed to the decomposition of potato straw increasing soil organic phosphorus content, thereby providing abundant substrates for acid phosphatase, stimulating its synthesis and secretion, and ultimately accelerating the conversion of organic phosphorus to available phosphorus (Yang et al., 2023a). Polyphenol oxidase is typically involved in the decomposition of recalcitrant organic compounds such as lignin; the lignin-rich composition of potato straw likely stimulated soil microorganisms to synthesize this enzyme, resulting in a significant increase in its activity (Liu et al., 2023a).

### 4.2 Effects of potato straw incorporation on soil microbial community structure and function

Building on the nutrient changes described above, potato straw incorporation also profoundly restructured the soil microbial community. The soil microbial community serves as the core driver of soil ecosystem functioning, and shifts in its structure and function directly influence soil nutrient cycling and plant growth (Zeng et al., 2024). In the present study, analysis of soil microbial alpha diversity following potato straw incorporation revealed a significant increase in the Sobs and Chao1 indices of the bacterial community, whereas all fungal diversity indices showed non-significant increases. These results suggest that straw incorporation provided abundant carbon and nitrogen sources for soil microorganisms, improved the soil microenvironment, and created more favorable conditions for bacterial growth and reproduction, thereby enhancing the species richness and diversity of the bacterial community. In contrast, the fungal community exhibited a comparatively modest response to straw incorporation, possibly because fungal decomposition of straw predominantly targets recalcitrant components at later stages, resulting in no significant improvement in diversity over the short term (Zhao et al., 2019; Wang et al., 2021a).

At the bacterial phylum level, straw incorporation significantly increased the relative abundances of *Acidobacteriota, Gemmatimonadota, Verrucomicrobiota*, and *Firmicutes*, while reducing the proportions of *Bacteroidota* and *Cyanobacteria*. *Acidobacteriota* predominantly comprises acidophilic, oligotrophic microorganisms (Fierer et al., 2007). The slight increase in soil pH following potato straw incorporation likely suppressed extremely acidophilic subgroups (e.g., Gp1) while simultaneously creating more favorable competitive conditions for subgroups adapted to near-neutral pH ranges (e.g., Gp6), resulting in community-level succession at the subgroup level and the maintenance of relatively high total abundance at the phylum level (All-Russian Research Institute For Agricultural Microbiology et al., 2019). *Firmicutes* harbors numerous functional strains capable of producing cellulases and amylases, enabling efficient decomposition of carbohydrates and other components in straw, and was thus significantly enriched following straw incorporation (Huang et al., 2023). *Gemmatimonadota* and *Verrucomicrobiota* are involved in soil carbon and nitrogen cycling, and their increased abundances further corroborate the enhanced soil nutrient transformation capacity conferred by straw incorporation (Yuan et al., 2023; Yue et al., 2023).

At the fungal phylum level, straw incorporation led to significant increases in *Chytridiomycota* and *Glomeromycota*, alongside a significant decrease in *Basidiomycota*. Arbuscular mycorrhizal fungi (AMF) within Glomeromycota can form symbiotic associations with plant roots and are associated with the uptake of phosphorus, nitrogen, and other nutrients, potentially through expansion of the root absorption area and secretion of organic acids; consistent with the AMF enrichment observed in the present study, this is likely to have contributed to soybean growth promotion (Wang et al., 2023). Certain taxa within *Chytridiomycota* participate in organic matter decomposition and plant disease suppression, and their increased abundance may be associated with improvements in the soil micro-ecological environment following straw incorporation (Wu et al., 2026). No steroidal glycoalkaloid or annotated degradation product met the predefined identification and differential-abundance criteria. This result should be interpreted in light of the methodological differences between untargeted and targeted metabolomics. Untargeted metabolomics is a discovery-oriented approach that profiles a broad range of metabolites but is not specifically optimized for every compound class, whereas targeted metabolomics measures predefined compounds using optimized LC–MS/MS parameters and authentic standards, thereby providing higher sensitivity and specificity. Because the present study relied on untargeted profiling and no targeted standard-based assay was performed, the non-detection of steroidal glycoalkaloids cannot be interpreted as evidence of their absence. The unchanged fungal alpha diversity indicates stable within-sample richness and evenness, while shifts in selected fungal taxa indicate compositional reorganization rather than a community-wide diversity loss.

LEfSe analysis further revealed the selective enrichment of microbial functional groups in response to straw incorporation. Among bacteria, straw incorporation significantly enriched taxa associated with straw degradation, including *Firmicutes* (represented by *Paenibacillus*), as well as *Gemmatimonas* and *Nitrosomonadaceae*, which are linked to carbon, nitrogen, and phosphorus cycling (Huang et al., 2023; Yuan et al., 2023; Yue et al., 2023). In contrast, the non-incorporation treatment enriched taxa involved in the decomposition of recalcitrant organic matter and adaptation to carbon stress, such as the *Allorhizobium–Neorhizobium–Pararhizobium–Rhizobium* complex (rhizobial complex group). The absence of this enrichment under straw incorporation may be associated with a shift in the soil microbial niche away from rhizobium-favored symbiosis and toward a multi-component system of AMF and PGPR (e.g., *Paenibacillus*) where AMF facilitate phosphorus acquisition and PGPR mediate nitrogen fixation and hormone production, together replacing part of the rhizobial functions. Among fungi, straw incorporation significantly enriched communities dominated by AMF and biocontrol fungi (biocontrol-associated Ascomycetes, e.g., *Hypocreales*), whereas the non-incorporation treatment enriched saprotrophic and potentially pathogenic fungi, including *Rhizoctonia* and *Cladosporium*. This may be attributed to the fact that straw incorporation not only altered microbial community structure but also optimized microbial functional composition by enriching functionally dominant taxa, thereby reinforcing key ecological functions such as straw degradation, nutrient cycling, and disease suppression, and ultimately establishing a healthier rhizosphere micro-ecological environment for soybean growth (Liu et al., 2025).

### 4.3 Effects of potato straw incorporation on differentially accumulated soil metabolites

The restructuring of the microbial community further drove changes in soil metabolite composition. Soil metabolites, functioning as “signaling molecules” and “functional carriers” mediating interactions among microorganisms, plants, and the soil environment, directly reflect the intensity of metabolic activity and the direction of material transformation within the soil ecosystem (Song et al., 2024; Ku et al., 2025) In the present study, lignans, phenolic glycosides, and flavonoids were found to be significantly upregulated following straw incorporation, whereas organoheterocyclic compounds were significantly downregulated. Furthermore, the two sample groups exhibited clear separation in the PLS-DA plot, indicating that straw incorporation substantially remodeled soil metabolite composition. From the perspective of metabolite classification, flavonoids and phenolic glycosides not only possess antioxidant and antimicrobial properties, but are also associated with plant–microbe symbiotic signaling and with soil fertility improvement, potentially through nutrient chelation and soil structure amelioration. (Hassan and Mathesius, 2012; O'Banion et al., 2020). Lignans are closely associated with the decomposition and transformation of straw lignin, and their upregulation indicates enhanced lignin degradation metabolic activity following straw incorporation, consistent with the significant increase in polyphenol oxidase activity reported previously.

KEGG pathway enrichment analysis revealed that the differentially accumulated metabolites were significantly enriched in pathways including the biosynthesis of histidine- and purine-derived alkaloids, biosynthesis of plant secondary metabolites, arachidonic acid metabolism, and biosynthesis of phenylpropanoids. The enrichment of plant secondary metabolite biosynthesis observed in the present study suggests enhanced plant stress tolerance and indicates a capacity to modulate soil microbial community structure, fostering beneficial rhizosphere interactions (Hu et al., 2018). Lipid metabolism-related pathways were significantly enriched following straw incorporation. The enrichment of arachidonic acid metabolism, which is closely associated with lipid metabolism, suggests that straw return may be associated with nutrient storage and release in soil, potentially through its association with soil microbial lipid metabolic capacity (Chen et al., 2022; Liu et al., 2023b). As precursors of lignin biosynthesis, phenylpropanoids, through the enrichment of their biosynthetic pathway, further corroborate the transformation and utilization of lignin during straw decomposition, thereby providing a material foundation for soil organic matter accumulation (Thirunavukkarasu et al., 2025).

In summary, straw incorporation remodeled soil metabolome composition and activated key metabolic pathways—including biosynthesis of plant secondary metabolites, the arachidonic acid metabolism, and biosynthesis of phenylpropanoids—thereby providing metabolic-level support for the optimization of soil ecosystem functioning.

### 4.4 Interaction network among microorganisms, metabolites, and environmental factors

In soil ecosystems, complex interactions occur among soil physicochemical factors, microbial communities, and metabolites, which jointly regulate soil fertility and plant growth (Hu et al., 2018). In this study, Spearman correlation and co-occurrence network analyses were used to reveal the interaction patterns among these three components following potato straw incorporation. Correlation analysis showed that the bacterial community could be divided into two core groups with significantly different metabolic preferences: Proteobacteria-affiliated taxa preferentially utilized readily degradable carbon sources such as organic acids and participated in the rapid turnover of soil carbon (Hacquard et al., 2015), whereas taxa such as *Gemmatimonadota* and *Firmicutes* were more efficient at utilizing nitrogen-containing compounds such as aromatic amino acids and participated in soil nitrogen metabolism (Trujillo et al., 2015). The fungal community was likewise divided into two branches that were closely associated with fungal functional traits: in Branch 1, AMF and biocontrol fungi (e.g., *Trichoderma*) may be associated with plant symbiosis and disease suppression, potentially through their association with the synthesis or transformation of flavonoids (Liu et al., 2025; Zhao et al., 2025), whereas the saprotrophic fungi in Branch 2 preferentially used terpenoids and organic acid derivatives as carbon sources and participated in organic matter decomposition. Co-occurrence network analysis further showed that environmental factors such as pH, organic matter (OM), alkali-hydrolyzable nitrogen (AN), and available phosphorus (AP) were highly connected node in the exploratory network of the interaction network, forming dense positive and negative correlation links with microbial communities and metabolites (Yang et al., 2024). Notably, pH and organic matter exhibited high degree centrality in both the bacterial and fungal networks; they were positively correlated with each other, positively correlated with xanthosine and Sediminibacterium, and negatively correlated with urocanic acid, consistent with their role as an “variable centrally positioned in the correlation network for community assembly and metabolite retention.”

From a metabolic-pathway perspective, citrulline—an intermediate of the arginine–urea pathway—was associated with the Rhizobium complex and Pseudorhodobacter; L-histidine and urocanic acid represent two consecutive steps of the histidine degradation pathway, and the latter was significantly negatively correlated with pH, suggesting that this pathway is highly sensitive to changes in soil pH. The catabolism of purine nucleosides such as *xanthosine* proceeds through a xanthine intermediate to ultimately produce allantoin; this conversion is particularly important in soybean, because soybean is a legume that relies on ureides (allantoin and allantoic acid) as its primary form of nitrogen transport (Werner and Witte, 2011; Collier and Tegeder, 2012). The simultaneous detection of these three nitrogen metabolic pathways indicates that potato straw incorporation broadened the soil organic nitrogen substrate spectrum rather than merely enlarging a single nitrogen pool, a conclusion corroborated by the concurrent increases in alkali-hydrolyzable nitrogen content and urease activity. As a key soil enzyme, urease (S-UE) was significantly positively correlated with flavonoid metabolites such as daidzein and genistin and with *Plenodomus* fungi, indicating that urease not only participates directly in soil organic nitrogen mineralization but may also help coordinate rhizosphere microecological balance through its interactions with functional microorganisms and rhizosphere metabolites (Li Y. et al., 2023). In addition, acid phosphatase (S-ACP) was positively correlated with *Ganoderma* and negatively correlated with *Pseudopithomyces*, in agreement with previous studies (Lu et al., 2022; Ji et al., 2024) further suggesting that soil enzymes may represent linking nodes between microbial taxa and metabolites in the co-occurrence network. A recent 10-year field study combined metagenomics and rhizosphere metabolomics to link straw management with carbon-cycling genes, soil carbon fractions, and annual crop productivity (Yang et al., 2025)). That design provides direct functional and long-term evidence that is not available in the present one-season amplicon-based pot experiment; our network results should therefore be treated as hypotheses for comparable field and functional validation.

This study has four design limitations. First, the field-embedded pots restricted root expansion and altered vertical water and nutrient transport relative to an open field. Second, only one straw incorporation rate and one growing season were evaluated, so dose-response relationships, optimal application rates, cumulative effects, and possible pathogen accumulation could not be assessed. Third, rhizosphere soil was sampled only at harvest, which prevents separation of transient decomposition effects from end-point accumulation. Fourth, the untargeted LC-MS workflow was not designed for targeted quantification of potato steroidal glycoalkaloids or their degradation products. Accordingly, the observed microbial and metabolite patterns are interpreted as harvest-stage associations, and the agronomic results are limited to measured traits and yield components. Multi-rate, multi-season field experiments with repeated sampling, direct grain-yield and biomass measurements, targeted glycoalkaloid analysis, and functional-gene validation are required before field-scale mechanisms or yield benefits can be concluded.

## 5 Conclusions

Potato straw incorporation significantly increased soil nutrient contents—including organic matter, available potassium, available phosphorus, and alkali-hydrolyzable nitrogen—and enhanced acid phosphatase and polyphenol oxidase activities, thereby improving the soil fertility base. Meanwhile, straw incorporation reshaped the soil microbial community toward nutrient-cycling and symbiotic growth-promoting functions, promoting the enrichment of beneficial axa putatively associated with the stated function such as *Paenibacillus*, Nitrosomonadaceae, and arbuscular mycorrhizal fungi; it also markedly altered soil metabolite composition, reflecting active synthesis of flavonoids and phenolic compounds, active phenylpropanoid metabolism, and the activation of multiple organic nitrogen transformation pathways, thereby broadening the soil organic nitrogen substrate spectrum. Soil pH, organic matter, urease activity, and soybean isoflavone metabolites exhibited high degree centrality in the co-occurrence network, suggesting a multi-node co-occurrence pattern among soil physicochemical factors, enzyme activities, microbial communities, and metabolites. This interaction network synergistically enhanced nutrient-transformation efficiency and promoted the enrichment of beneficial functional microorganisms, thereby optimizing rhizosphere ecosystem function and ultimately promoting soybean growth and yield components formation. Further multi-year field studies with temporal sampling, multiple application rates, and functional validation are required to determine the underlying ecological mechanisms and agronomic relevance of potato straw incorporation. This study provides a theoretical basis for straw resource utilization and cultivated-land quality improvement in the potato–soybean rotation system.

## Data Availability

The datasets presented in this study can be found in online repositories. The names of the repository/repositories and accession number(s) can be found below: https://www.ncbi.nlm.nih.gov/, PRJNA1493838 https://doi.org/10.21228/M8128X, ST005041.
