# Supplementary material for: Potato straw incorporation improves soybean agronomic traits and yield components and is associated with shifts in rhizosphere soil properties microbial communities and metabolites
Source: Front Microbiol. 2026 Oct 14;17:1990822. doi: 10.3389/fmicb.2026.1990822 (PMC13713313; doi:10.3389/fmicb.2026.1990822)
Supplement: Supplementary file 1 [file Supplementary_file_1.pdf]

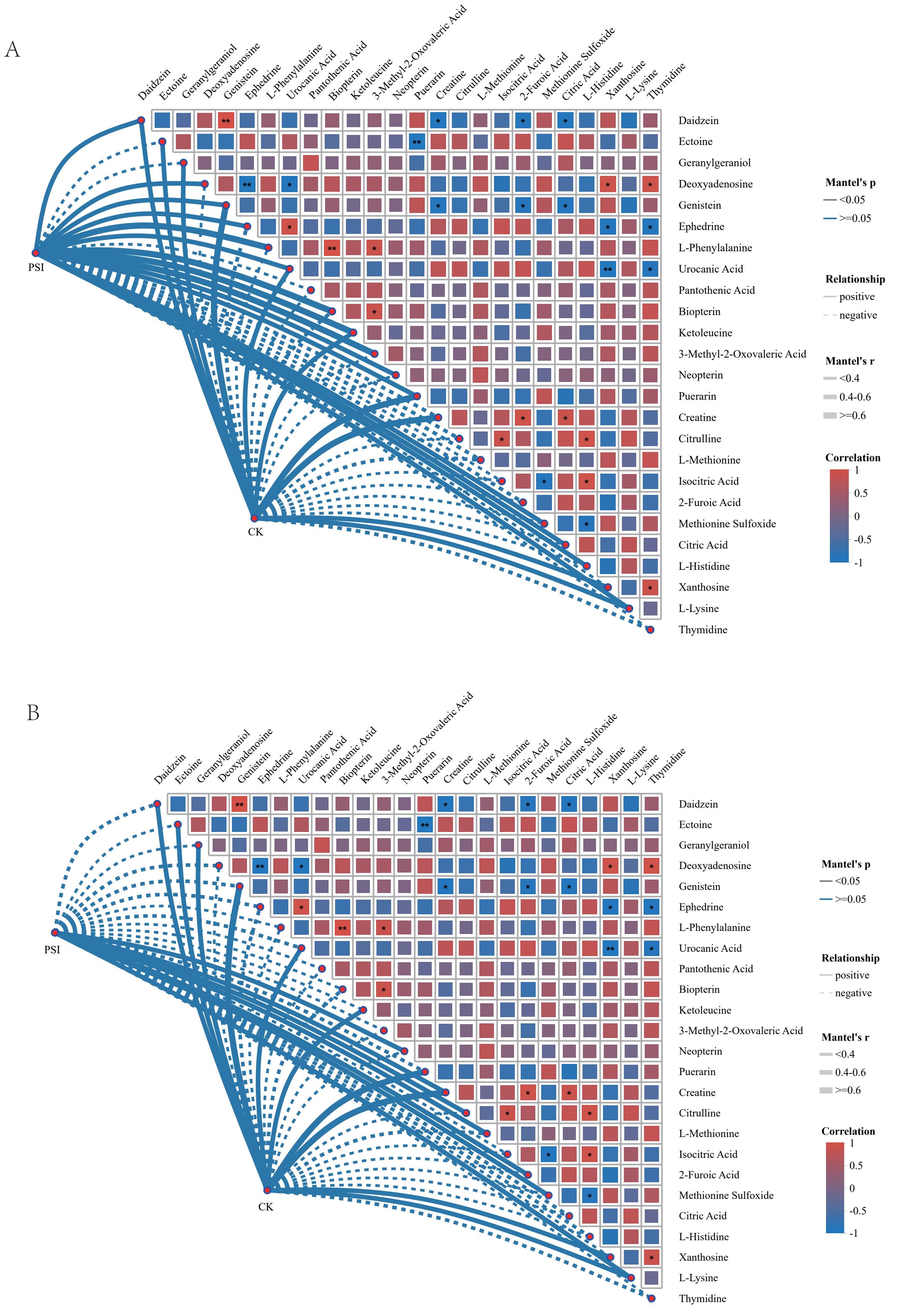

Figure A1. Mantel test heatmaps showing correlations between treatments (CK vs. PSI) and the overall rhizosphere metabolite profile based on Bray-Curtis distance matrices.
(A) Bacterial community. (B) Fungal community. Edge width corresponds to Mantel's r (absolute value), with solid lines indicating $p < 0.05$ and dashed lines indicating $p \geqslant 0.05$. The heatmap shows correlations among metabolites, with red indicating positive correlations and blue indicating negative correlations; darker colors represent stronger correlations. Asterisks denote statistical significance: * $0.01 < p \leqslant 0.05$, ** $0.001 < p \leqslant 0.01$, *** $p \leqslant 0.001$. CK, control (without straw return); PSI, potato straw incorporation.
